# Beyond the spotlight: Unveiling the gender bias curtain in movie reviews

**DOI:** 10.1371/journal.pone.0316093

**Published:** 2025-01-29

**Authors:** Jad Doughman, Wael Khreich

**Affiliations:** Suliman S. Olayan School of Business (OSB), American University of Beirut (AUB), Beirut, Lebanon; Federal University of the ABC: Universidade Federal do ABC, BRAZIL

## Abstract

Historically, the film industry has been male-dominated both in front of and behind the camera, resulting in a longstanding gender imbalance in storytelling and representation. This legacy of male-centric narratives may unconsciously influence critics’ expectations and judgments. Existing literature suggests that negative critiques in movie reviews can significantly impact actors’ earnings by diminishing a film’s commercial prospects. This influence extends to potential reductions in back-end compensation and marketability for future projects, and it can directly affect actors’ well-being, leading to increased stress levels and elevated cardiovascular reactivity. Previously, gender biases in movie reviews were computed using disparities in male-led versus female-led movie ratings or box-office earnings; however, no work has been done to quantify the linguistic biases within movie review transcripts. This work aims to leverage our language-model-powered gender bias detection system to measure benevolent sexism, hostile sexism, explicit marking of sex, dehumanization, and generic pronouns in reviews published by professional critics. Therefore, we analyzed 17,165 professionally written reviews, comprising a total of 735,000 sentences. Our analysis uncovers pronounced representation bias in key movie roles, with 72% of first actors, 91% of first directors, and 86% of first writers being male. More importantly, the findings indicate that movies with female-dominated casts evoke, on average, 149% higher magnitude of hostile sexism and 44% higher magnitude of benevolent sexism in their reviews compared to movies with male-dominated casts. We also find that benevolent sexism is most common in Family and Music genres, reflecting the romanticization of gender roles, whereas hostile sexism peaks in Romance. A non-parametric statistical analysis revealed significant gender differences in benevolent and hostile sexism scores for movie reviews, with female first actors, directors, and writers receiving higher levels of both benevolent and hostile sexist criticism compared to their male counterparts.

## 1 Introduction

As early as the age of five, a child can recognize their inherent membership within a set of groups in society [1, 2]. As a child grows and entrenches themselves further within their local neighborhood, school, and nation; they develop “fierce in-group loyalties” amongst a variety of gender, ethnic, and racial groups [1, 2]. Some psychologists believe that children are “rewarded” by virtue of their membership and that the “reward” yields their loyalty [1, 2]. Allport hypothesized that the sheer “separation of human groups” prompts the psychological processes that result in prejudicial behavior [1, 2].

Gender bias can be relayed in language in a variety of ways, however, in its reduced form, we define it as being an exclusionary, implicitly prejudicial, or generalized representation of a specific gender or social group as a function of various societal stereotypes [3]. Other than the direct implications of biased language such as making people feel misunderstood, cast out, or misrepresented, it also has macro-societal impacts on labor force participation, children’s mental imagery, and career attractiveness [4–9].

Historically, the film industry has been male-dominated both in front of and behind the camera, resulting in a longstanding gender imbalance in storytelling and representation. This legacy of male-centric narratives may unconsciously influence critics’ expectations and judgments, which in turn influences actors’ earnings by diminishing a film’s commercial prospects and directly impacting the marketability for future projects [10]. Antagonistic reviews, especially from critics, can have direct psychological implications on the targeted cast members, leading to heightened stress levels and increased cardiovascular reactivity [11, 12].

Existing works related to measuring biases in the film industry are geared towards gaps between male and female-led movie ratings or divergent personality traits in male and female movie characters [13–15]. However, no work has been done to quantify linguistic biases within professional movie review transcripts and analyze disparities in review antagonism for male-led vs female-led movies.

To address this gap, we construct a metadata-enriched, professionally critiqued movie review dataset. We start with the Movie Review Data [16] and augment it with relevant metadata using the Open Movie Database (OMDb) API, a RESTful web service to obtain movie metadata, including the film rating. According to the Motion Picture Association (MPA), ratings include G (General Audiences), PG (Parental Guidance), PG-13 (Parents Strongly Cautioned), R (Restricted), and NC-17 (Adults Only), along with information on genre, release year, and the names of directors, writers, and actors. We use an off-the-shelf gender name identifier to predict the gender of the first actor, writer, and director for each movie based on their first names. For names that yielded ambiguous results, such as ‘mostly male’ or ‘mostly female,’ we implemented a manual verification process using Wikipedia. Finally, we propose a metric called the Gender Representation Disparity Index (GRDI), devised to measure the disparity in gender representation across various roles—director, writer, and actor—in each movie.

In an effort to measure various linguistic biases in review transcripts, we employ a gender bias detection system, which classifies biases into categories such as benevolent sexism, hostile sexism, and dehumanization, leveraging the previously proposed taxonomy [17]. The system, comprised of a pool of fine-tuned language model classifiers, is trained on extensive datasets from diverse platforms such as Quora and Twitter, annotated by experts who demonstrate high agreement in their evaluations [17]. We start by tokenizing each transcript of the movie review. Then, we pass each sentence through the system for inference and record the resulting bias scores [17].

We leverage the constructed dataset along with the bias scores assigned to each review sentence to quantify bias in the film industry in two ways: (1) by analyzing the disparity in role representation and (2) by examining the differences in antagonism or bias expressed in movie review texts. Our analysis revealed that women were significantly underrepresented in major film roles, with 72% of first actors, 91% of first directors, and 86% of first writers being male. Temporally, female representation among first actors increased only marginally, from 23% in 1994 to 31% by 2001. The representation of female directors only increased from 3% to 10% in the same period. Female writers hovered around 13%-16%, showing minimal progress, too. Additionally, films with predominantly female casts were subject to 44% more benevolent sexism and a 149% increase in hostile sexism compared to those with all-male casts. This trend was consistent across different genres, with certain genres, like Romance, exhibiting even higher levels of sexism in review texts. Finally, we observed that as the GRDI shifts from an all-male cast (0.00) to an all-female cast (1.00), explicit sex markers in reviews decrease by 46.52%. This decline is likely due to the prevalence of male-specific markers like ‘businessman’ and ‘cameraman’ in films with predominantly male casts.

Our work underscores the pervasive prejudice in movie reviews, particularly in portraying and critiquing female-led films. By raising awareness about these subjective biases among critics, we aim to mitigate their negative impacts and promote a more balanced and equitable representation in the film industry.

## 2 Background

### 2.1 Biases in movie ratings

Recent studies have highlighted the multifaceted influences on movie ratings and their impact on box office earnings, shedding light on prevalent biases. Fowdur et al. examined racial biases in newspaper critics’ movie reviews and their influence on viewer demand [13]. Analyzing data from 68 critics for 566 US movies (2003-2007), accounting for various factors, their study reveals that movies with a black lead actor and an all-white supporting cast receive ratings about 6% lower compared to movies without a Black lead actor and an all-White supporting cast [13]. This bias leads to potential revenue losses of up to 4% ($2.57 million average), suggesting that racial prejudice in role compositions impacts critic assessments and market results [13].

Similarly, Basuroy et al. investigated the influence of critics on film box office earnings, considering the roles of stars and budgets [14]. The authors analyze whether critics impact public decisions (influencers), predict decisions (predictors), or both [14]. They find that positive and negative reviews relate to weekly box office revenue over eight weeks, indicating dual roles. However, negative reviews’ impact wanes over time, favoring the influencer role [14]. Comparing positive and negative effects, films exhibit a negativity bias, particularly in the first week [14]. Stars and budgets moderate reviews; popular stars and big budgets boost revenue for films with more negative than positive reviews, less for the opposite [14]. The study extends prior research, guiding film studios in strategically managing reviews for revenue enhancement [14].

In contrast to the above findings, Lindner and Schulting investigated whether the presence of independent female characters, as assessed by the Bechdel Test, affects critic evaluations of films [18]. The Bechdel Test is an informal measure used to evaluate the representation of women in fiction, especially in movies. Named after cartoonist Alison Bechdel, the test requires a work to have at least two named female characters who engage in a conversation about something other than a man. Analyzing 975 widely distributed movies from 2000 to 2009, the study examines if films passing the Bechdel Test have different Metacritic scores. Control variables, including production label, genre, budget, star presence, and sequel status, are considered [18]. The findings indicate that the inclusion or absence of independent female presence does not statistically influence a film’s composite critical evaluation [18]. This suggests that while critical reviews do not significantly contribute to women’s under-representation in film, they also do not actively advocate for movies featuring independent female characters [18].

### 2.2 Bias in film scripts and subtitles

Several researchers have conducted in-depth analyses of gender biases and representations in movie subtitles and scripts. Haris et al. analyzed film scripts from various genres, transforming these scripts into vector representations or embeddings [15]. They identified prevalent patterns in the personality traits of male and female characters, which reinforce societal stereotypes [15]. Specifically, men are often depicted as more dominant and envious, while women are portrayed in more joyful roles [15]. The study introduces a new method for converting dialogues into an array of emotions, utilizing Plutchik’s wheel of emotions [15].

Similarly, Kagan et al. analyzed a century’s worth of film, examining gender bias in female on-screen characters [19]. The findings show a positive trend, with a steady increase in the presence of female roles and a growing number of films passing the Bechdel test [19]. Finally, Khadilkar et al. employed advanced natural language processing techniques to analyze English subtitles from 700 Bollywood films over seven decades, alongside Hollywood films and Academy Award nominees [20]. Their study revealed that although the depiction of women in movie dialogues has progressed over time in both Bollywood and Hollywood, significant gender bias persists, with a lack of gender parity [20]. They also identified a consistent bias in Bollywood towards fair skin across all examined periods. Furthermore, their geographical analysis showed a better representation of several Indian states over time but a persistent under-representation of many northeastern Indian states [20].

Xu et al. used word embedding techniques to identify the “Cinderella complex,” where female characters are depicted as emotionally dependent on males [21]. Covering 7,226 books, 6,087 movie synopses, and 1,109 movie scripts, the research found that male characters often have adventure-oriented roles, while females are portrayed in romantic contexts [21]. Istead et al. examined gender bias in movies by analyzing gendered language in over 40,000 movie subtitle files [22]. The study evaluates changes in bias based on factors like release year, genre, country of origin, MPAA rating, IMDb scores, and box office records. The findings reveal a significant male bias in over 75% of recent films [22].

Computational analyses reveal that certain professions, such as lawyers and doctors, are frequently depicted with polarized sentiment in media, suggesting an influence on public perception beyond just the depiction frequency [23]. Studies show that even minor roles in filmmaking reflect gender disparities rooted in historical practices, echoing broader professional trends across time and media [24]. These studies underscore the need for more equitable representation and systemic changes to address gender biases in both media portrayals and professional environments.

As highlighted above, existing literature has been geared primarily towards rating or subtitle-centric analysis of bias with no focus on linguistic biases embedded within movie review transcripts, which drastically impacts the earnings of cast members and their psychological well-being. Additionally, there is a lack of unified datasets that include professionally critiqued movie reviews and metadata on the cast, including their genders. As a result, the below sections describe our dataset construction process and the gender bias classifiers utilized to measure bias within review transcripts.

## 3 Dataset construction

**Fig 1:**
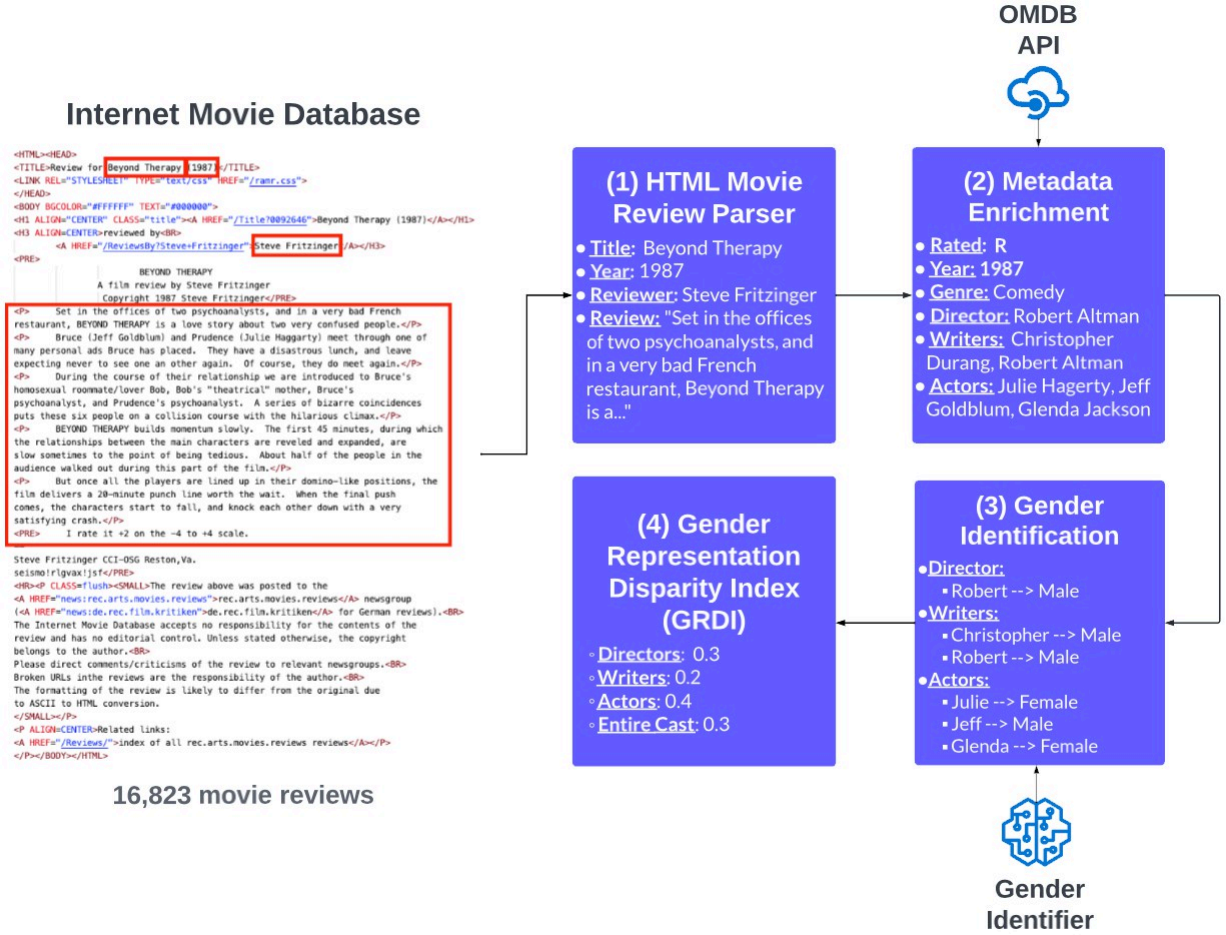
Dataset construction process, from parsing raw movie reviews in the movie review data, enriching metadata with the OMDb API, identifying genders of key contributors (actors, director, writer), to measuring gender representation metrics.

As far as we know, no professional critic movie review dataset currently includes metadata about the reviewed films, such as the names of the first writers, directors, and actors, as well as their genders. We focused on the first actors, writer, and director because they typically have the most significant impact on the film’s narrative and critical reception and are most affected by negative critiques. Including second, third, or additional actors, or even the entire cast, could dilute the gender bias signal, especially with a mixed-gender cast. Focusing on a single lead actor provides a more direct analysis of influence and impact. Therefore, we curated a dataset using the methodology presented in Fig 1 to investigate biases embedded within movie review text. This dataset will serve as the foundation for our case study, which aims to determine whether films featuring female protagonists tend to receive more negative criticism than those led by male protagonists. Such biases, if present, could potentially lead to lower box-office revenues and income disparities for the actors involved.

### 3.1 Dataset statistics

The Movie Review Data comprises of 17,165 critic reviews (2,544 unique movies) in the form of HTML files. Each HTML file contains the movie’s title, release year, reviewer name, and review text. Given the professional-critic nature of the reviews, each review transcript consists of an average of 40 sentences with a high emphasis on grammatical soundness, refer to Appendix A for an example.

**Fig 2:**
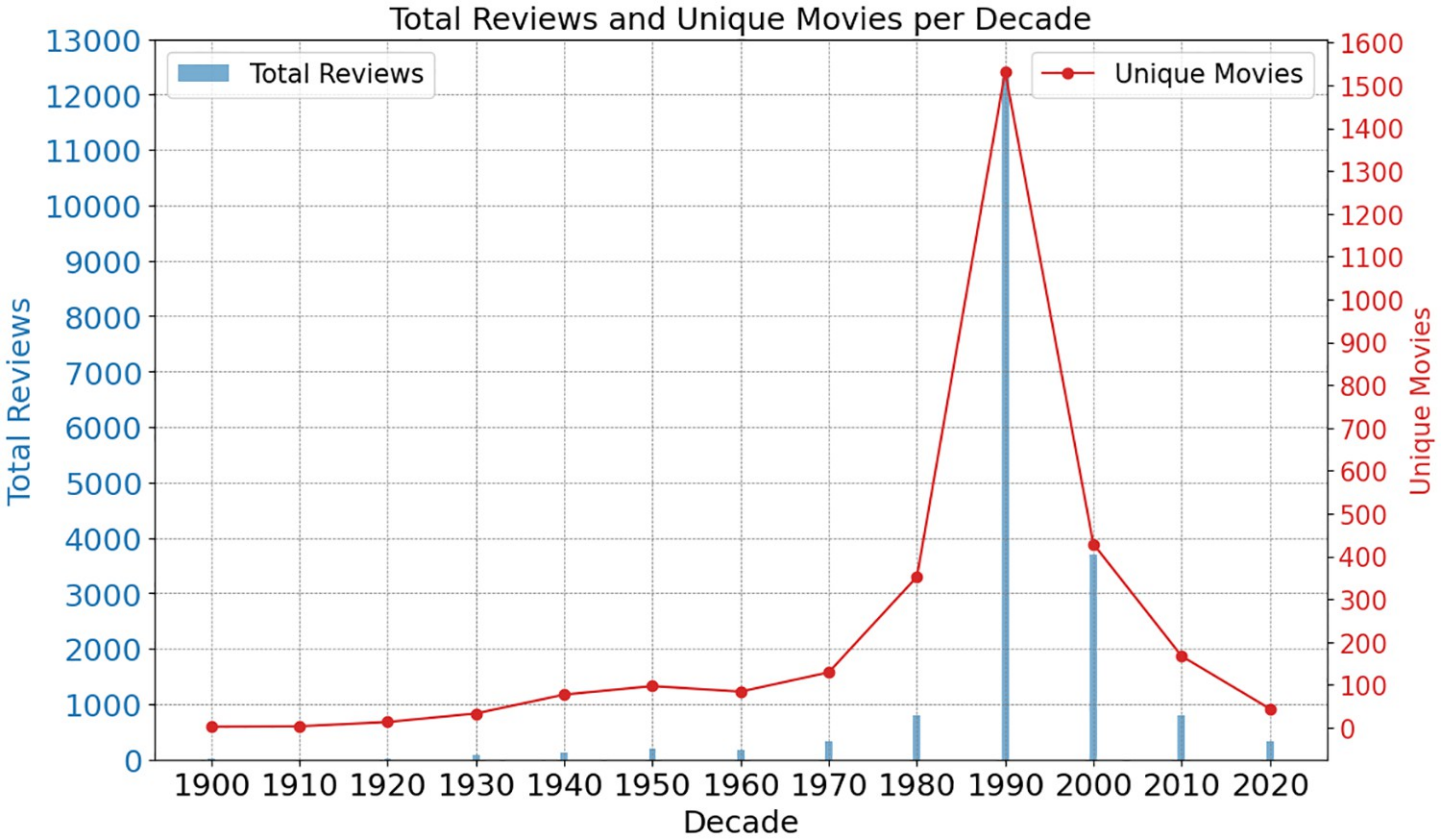
Total reviews and unique movies by decade. The bars indicate the number of reviews (left Y-axis), while the line graph shows the number of unique movies (right Y-axis).

As shown in Fig 2, the dataset spans from the 1900s to the 2020s. The distribution of reviews across decades is skewed towards more recent years, with the 1990s decade accounting for the largest number of reviews (12,306) across 1,532 unique movies. The 2000s and 1980s also have a substantial number of reviews at 3,708 and 795, respectively. Earlier decades like the 1910s and 1900s have very sparse data with only a few reviews each. Reviews from the 1900s, 1910s, 1920s, and 1930s were dropped due to having fewer than 120 reviews per decade, leaving a total of 17,165 reviews covering 2,544 unique movies. The dataset’s skew toward the 1990s reflects the original composition of the underlying professional review dataset, which we cannot control. Additionally, the reason we have more reviews than unique movies is that many films, particularly award winners, have multiple reviews from different critics in our dataset.

**Fig 3:**
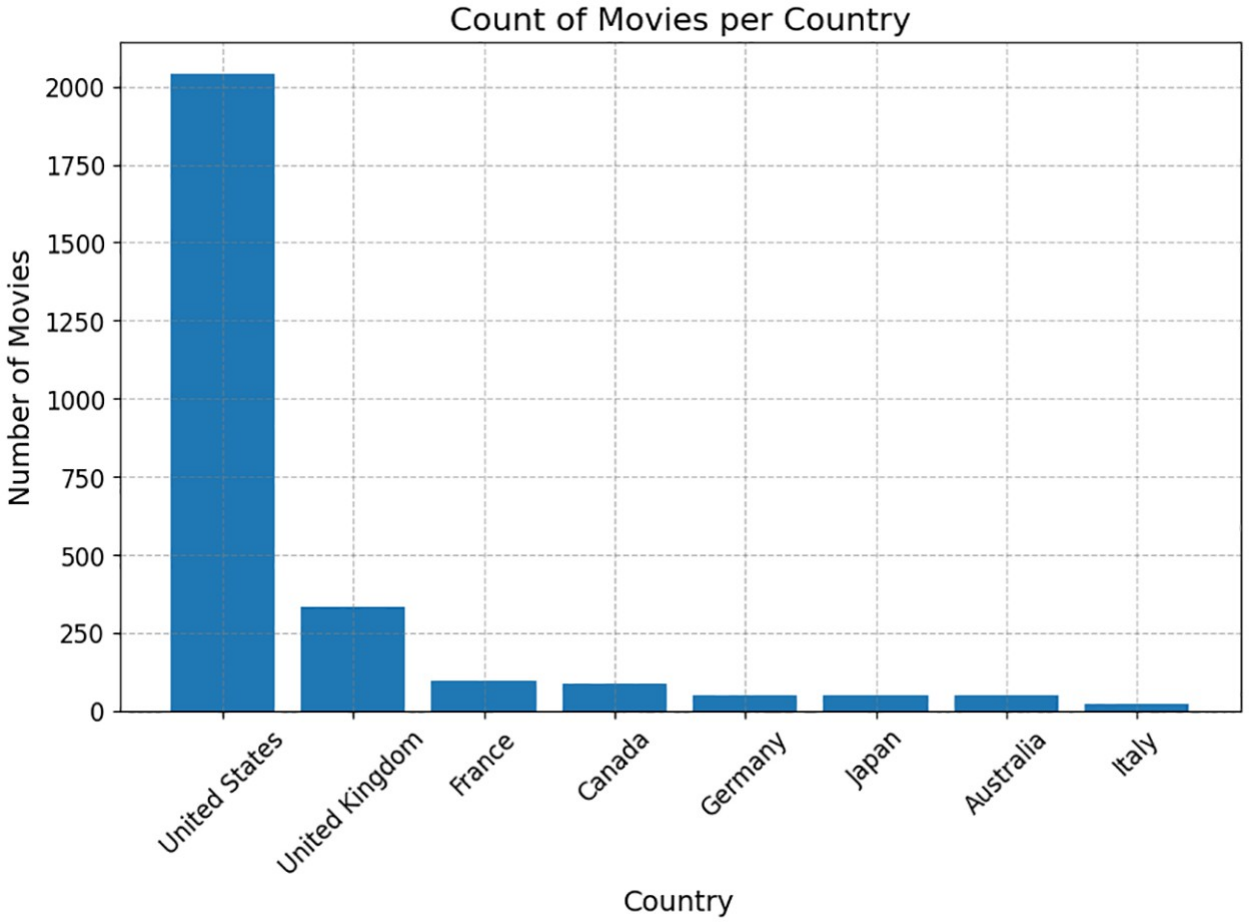
Distribution of movies by country.

As shown in Fig 3, the dataset primarily represents films from Western countries, with the United States dominating by contributing around 80% of the movies (2,041 films). The United Kingdom follows with 13% of total films. France, Canada, and Germany also contribute, but each with less than 100 movies. Appendix B provides examples demonstrating different types of bias in reviews.

### 3.2 Metadata enrichment

The Movie Review Data doesn’t include information about the actors, directors, and writers starring in each reviewed movie. This limits our ability to evaluate the gender biases in female and male-led movies. Thus, we utilize OMD API [25] to retrieve each unique movie’s rating, year, genre, directors, writers, and actors. The OMD API takes a movie name as input and returns its metadata; however, to validate that the returned metadata is associated with the reviewed movie, we match the release year returned by the API with the year in the HTML review file. The movie reviews pertaining to unmatched release years were dropped from our dataset.

### 3.3 Actor/director/writer gender identification

Although the OMD API did enrich our dataset with the necessary metadata, it does not return the gender of each actor, director, or writer. Thus, we use an off-the-shelf gender name identifier to infer the genders of the first actor, director, and writer. The gender predictor takes a first name as input and attempts to identify its gender by returning one of four classes: male, female, mostly_male, mostly_female. The names of a male and female label were appended directly into our data frame, while names returning mostly_male, mostly_female were appended into a human evaluation pipeline. Since actors, directors, and writers are generally public figures, we used Wikipedia to extract and validate their genders.

### 3.4 Gender Representation Disparity Index

In this section, we present the Gender Representation Disparity Index (GRDI), a metric devised to measure the disparity in gender representation across various roles—director, writer, and actor—in each movie. We propose two variants of GRDI: an aggregated variant that considers the overall gender distribution across all specified roles, and a non-aggregated variant that considers each role separately. The formulas for computing the aggregated and non-aggregated GRDI are as follows:

$$\begin{array}{c} {\text{GRDI}}_{\text{movie}\_j}^{\text{agg}}=\frac{F_{\text{director},j}+F_{\text{writer},j}+F_{\text{actor},j}}{F_{\text{director},j}+F_{\text{writer},j}+F_{\text{actor},j}+M_{\text{director},j}+M_{\text{writer},j}+M_{\text{actor},j}} \end{array}$$ (1)

For the aggregated GRDI, ${\text{GRDI}}_{\text{movie}\_j}^{\text{agg}}$ represents the aggregated GRDI for the *j*^*th*^ movie. *F*_director,*j*_, *F*_writer,*j*_, and *F*_actor,*j*_ are the numbers of females in the roles of director, writer, and actor, respectively, in the *j*^*th*^ movie. Similarly, *M*_director,*j*_, *M*_writer,*j*_, and *M*_actor,*j*_ represent the counts of males in these roles.

$$\begin{array}{c} {\text{GRDI}}_{\text{role},j}^{\text{non-agg}}=\frac{F_{\text{role},j}}{F_{\text{role},j}+M_{\text{role},j}} \end{array}$$ (2)

For the non-aggregated GRDI, ${\text{GRDI}}_{\text{role},j}^{\text{non-agg}}$ represents the non-aggregated GRDI for the given role in the *j*^*th*^ movie, and *F*_role,*j*_ and *M*_role,*j*_ are the counts of females and males, respectively, in that role for the *j*^*th*^ movie.

A GRDI value close to 0 indicates a male-dominated representation, while a value near 1 signifies a female-dominated representation. A value of 0.5 represents gender parity in the roles for that particular movie. In our analysis, we employ both the aggregated and non-aggregated variants of GRDI to comprehensively examine gender representation disparities across different roles and in the overall context of each movie.

## 4 Gender bias detection and mitigation system

The below section leverages a previously published gender bias taxonomy, dataset, and detection system to measure various forms of biases in movie reviews [17]. Here, we aim to provide an overview of the system, emphasizing its data-centricity, which offers analytical diversity in our case study.

### 4.1 Gender bias definition

We define gender bias in texts as an exclusionary, implicitly prejudicial, or generalized representation of a specific gender as a function of various societal stereotypes [3]. The sections below provide a bottom-up overview of gender bias, from its societal origin to its spillover onto language, while highlighting its perceptual and societal implications [3].

### 4.2 Societal origin

According to the social role theory, gender stereotypes originate from distinct roles traditionally assigned to men and women [26]. Historically, men and women have occupied varied roles, with men often involved in tasks demanding “speed, strength, and long absences from home,” while women typically focused on “domestic responsibilities, including child care” [27]. This division leads to several effects. First, men are generally seen and expected to be more agentic, characterized by being proactive, independent, and decisive, whereas women are often viewed and expected to be communal, embodying attributes like kindness, helpfulness, and benevolence [27]. Second, this division encourages men and women to develop specific skills and adapt their social behavior to meet the expectations of their roles [27]. Actors and observers in society tend to adopt traits associated with their respective roles [28]. This situation forms a self-reinforcing cycle that perpetuates gender biases, categorizing each gender into stereotypical roles and reinforcing these roles through the strive for role fulfillment.

### 4.3 System overview

**Fig 4:**
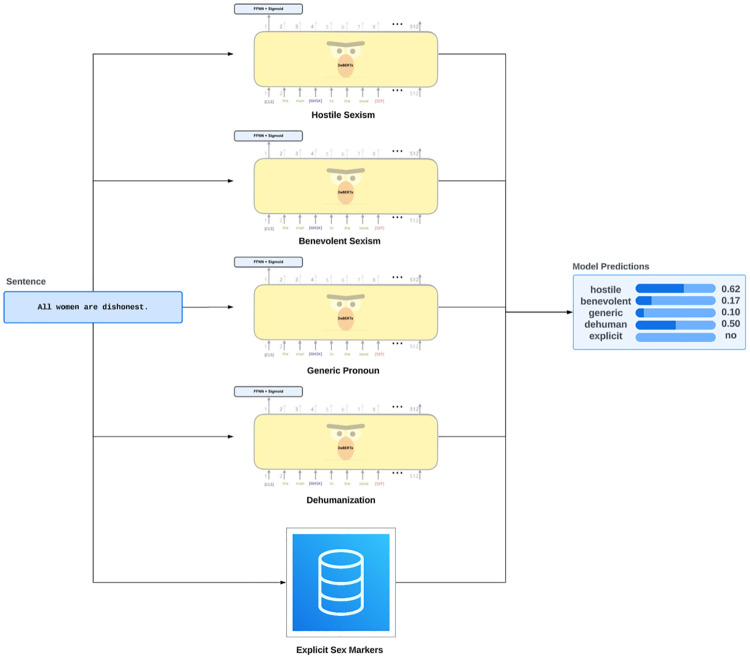
Inference pipeline of fine-tuned DeBERTa models, providing the likelihood of specific bias types in sentences and determining the presence or absence of bias for explicit sex markers.

Fig 4 provides an overview of the gender bias detection system used in this work based on the previously published taxonomy. We assume the following gender bias types: benevolent sexism, hostile sexism, dehumanization, generic pronouns, and explicit sex marking [3].

The diversity in the semantic and syntactic structure of each bias type offers analytical diversity as we cover a wide range of bias types [3]. Table 1, adopted from [3], provides an overview of the taxonomy with examples of each bias type. Hostile sexism is expressed in a negative, blatant, and aggressive manner, and it reflects men’s hatred toward women [29]. On the other hand, benevolent sexism is a form of gender bias characterized by seemingly positive attitudes and behaviors that idealize women as fragile and in need of protection while reinforcing traditional gender roles and male dominance [29]. Hostile sexism has a severe direct impact on women’s physiological well-being, such as increased stress levels, anger, and elevated cardiovascular reactivity [30]. Despite its seemingly well-intentioned nature, benevolent sexism can restrict women’s autonomy and contribute to gender inequality [31]. Dehumanization is the act of treating women as less than human, often by objectifying, stereotyping, or denying their agency and individuality [32]. These perceptions can contribute to objectification and aggressive behaviors against women [33]. Further examples from the movie reviews are presented in Appendix B.

**Table 1:** Categorization of gender bias in text, detailing subtypes such as generic pronouns, sexism, and exclusionary terms, with examples illustrating their manifestation in language. Adapted from [3].

Bias Type	Bias Subtype	Example
**Generic Pronouns**	Generic He	A programmer must carry his laptop with him to work.
	Generic She	A nurse should ensure that she gets adequate rest.
**Sexism**	Hostile Sexism	Women are incompetent at work.
	Benevolent Sexism	They’re probably surprised at how smart you are, for a girl.
**Exclusionary Terms**	Explicit Sex Marking	Chairman, Businessman, Manpower, Cameraman
	Neologisms	Man-bread, Man-sip, Man-tini

The training data used to train the system are drawn from a diverse set of sources, including platforms such as Quora, Twitter, and QuoteMaster [34–37]. These sources represent a broad spectrum of user-generated content, allowing us to create a robust dataset that encompasses various types of bias [34–37]. To ensure the quality of our annotated dataset, nine graduate-level annotators were employed to label each sentence [34–37].

To train each of the five binary classifiers, we experimented with fine-tuning and evaluating various conventional and modern machine learning models for detecting bias in text using the labeled datasets (hostile sexism, benevolent sexism, and dehumanization) [17]. Fine-tuned DeBERTa stands out (across all bias types) as the top-performing model on the test set due to its enhanced attention mechanism and disentangled matrix multiplication tests [17]. The system, consisting of five binary classifiers, takes a sentence as input and outputs a score corresponding to each bias type, except the explicit sex markers classifier, which returns the presence or absence of bias.

## 5 Measuring biases in film industry

This section aims to analyze the disparity in gender representation across movie roles using our metadata-enriched dataset and quantify the discrepancy in linguistic biases in movie reviews for movies starring male and female actors/actresses. Hence, we categorize bias in the film industry into two forms: (1) disparity in role representation and (2) difference in antagonism/bias in movie review texts.

### 5.1 Gender bias in movie role representation

Given that our dataset now contains the gender of the first actor, director, and writer, we computed the proportion of representation of each gender across each of the three roles for all 2,300 movies in our dataset. The below sections analyze the temporal evolution of this representation disparity and its breakdown across genres.

#### 5.1.1 Disparity in role representation by year

**Fig 5:**
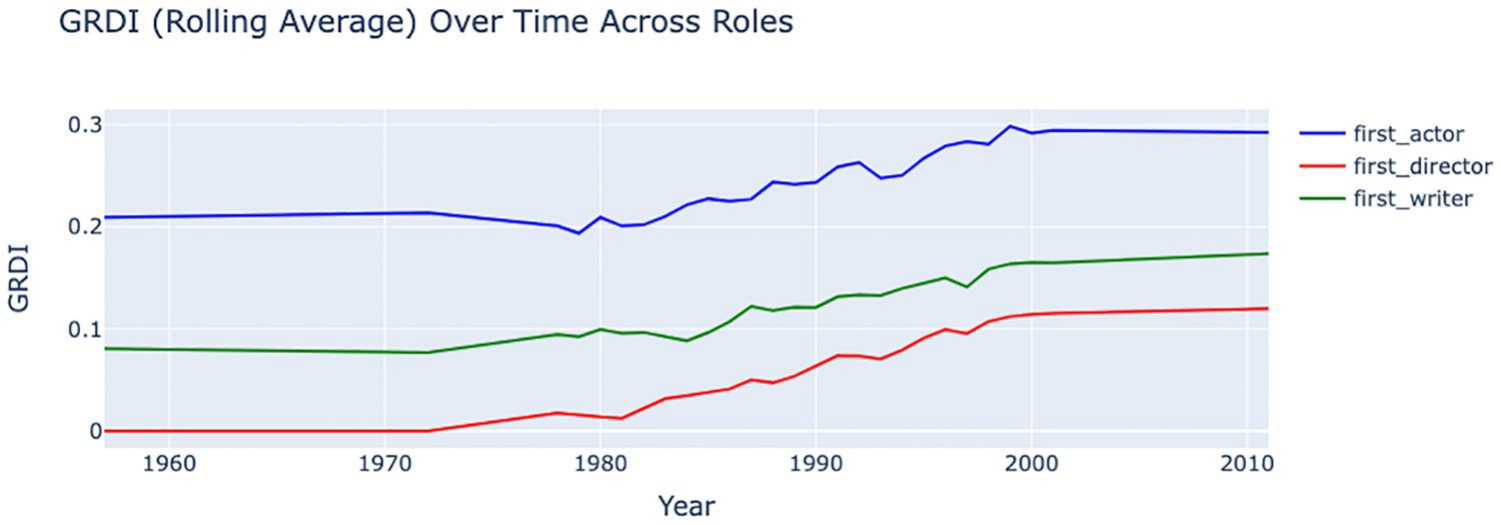
Temporal evolution of the Gender Representation Disparity Index (GRDI) from the 1990s to the 2020s, showing male-to-female representation proportions across actors, directors, and writers over time.

Reviewing the Gender Representation Disparity Index (GRDI) values over the years provides insights into the temporal evolution of gender representation trends in different roles, as shown in Fig 5. The first actor’s GRDI shows a steady increase, suggesting improvements in gender representation over the years, rising from approximately 10% female representation in 1960s to around 30% by 2010s, indicating a significant and continuous move towards equality in acting roles. In contrast, the first writer’s GRDI remains relatively low, increasing from about 10% female representation in 1960s to approximately 15% by 2010s. The first director’s GRDI also shows a gradual but more noticeable improvement, rising from about 5% in 1960s to close to 20% by 2010s. In summary, these trends reflect the slow and varying progress toward gender equality across different roles, each presenting unique representation and disparity patterns.

#### 5.1.2 Disparity in role representation by genre

**Fig 6:**
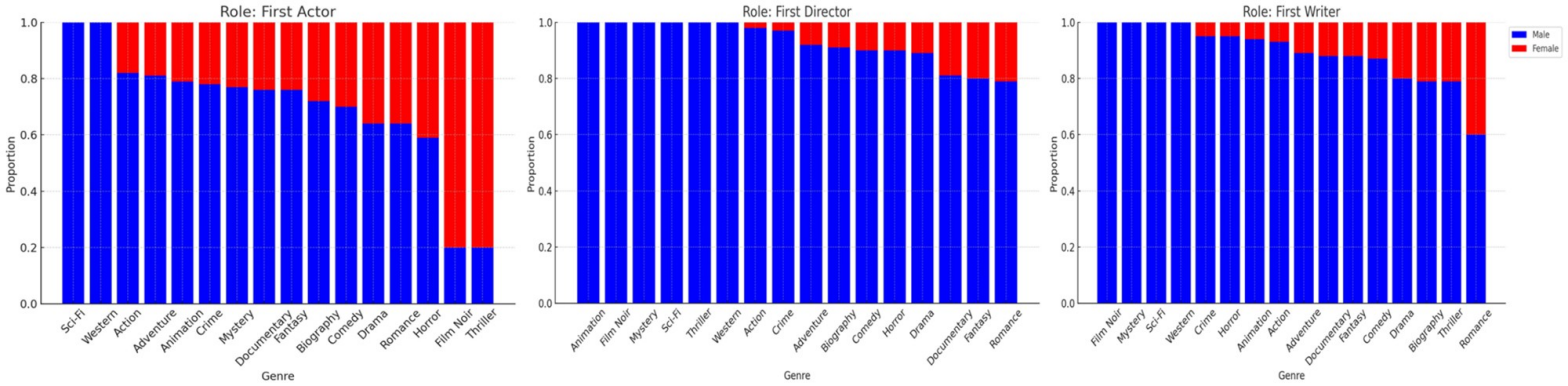
Clustered bar chart showing gender representation proportions across movie genres. Each cluster represents a genre, with bars for the first actor, director, and writer roles. Blue bars indicate male representation and red bars indicate female representation.

In analyzing gender composition within distinct film roles across various genres, a disparity between male and female representation was evident, as shown in Fig 6. For roles like the first actor, some genres, such as Sci-Fi and Western, were fully male-dominated, leaving females with no representation. There were only 2 (out of 16) genres that depicted a majority female representation in the first actor role, which are Film-Noir and Thriller, with proportions of 81% and 80%, respectively. This also can be considered as the portrayal of women in contemporary cinema often perpetuates a reductive and archaic narrative trope, predominantly casting them in roles that highlight objectification and romantic idealization. This trend not only undermines the multifaceted nature of female identity but also perpetuates a patriarchal perspective in popular culture. In the writer and director roles, no genre illustrated a majority of female representation, with the highest representation being 40% for Female Romance movie writers. All other genres illustrate an evident skew to male representation, especially directors and writers of Film-Noir, Mystery, Sci-Fi, and Western, which completely excluded females. The glaring discrepancy in gender representation underscores the crucial need for more inclusive and diversified participation across all facets of the film industry.

### 5.2 Gender bias in professional movie reviews

Other than the disparity in representation across movie roles, one hidden form of bias in the film industry pertains to the gap in antagonism within movie review manuscripts for movies starred and led by females versus males. Given that our text classifiers are trained on sentence-long inputs, not paragraph-long ones, we start by applying sentence tokenization to partition the reviews into a set of sentences while retaining a mapping to their original movie review IDs. The final data frame contains around 735,000 sentences. As shown in Fig 4, we pass each sentence into our detection system and return their probability scores. The probability scores are appended to our movie review data frame, which we then use for our analysis. The aim of quantifying biases in each sentence within movie reviews is to uncover any disparities in the critique of female-led and male-led movies. This section measures this disparity as a function of genres, roles, and various bias types.

#### 5.2.1 Disparity in gender bias by gender representation disparity index

**Fig 7:**
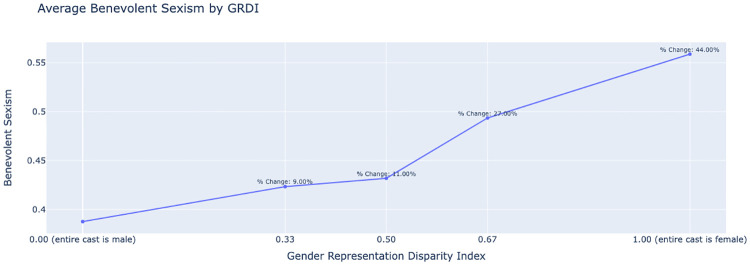
Relationship between the Gender Representation Disparity Index (GRDI) and average benevolent sexism in film criticism, illustrating increased sexism as the GRDI moves toward a female-dominated cast.

Fig 7 displays a positive correlation between the Gender Representation Disparity Index (GRDI) and average benevolent sexism in film criticism. The GRDI ranges from 0.00, indicating an all-male cast, to 1.00, denoting an all-female cast. As the GRDI increases, indicating a higher representation of females in the cast, the average benevolent sexism score also increases, which suggests that films with more women are subjected to higher degrees of benevolent sexism in movie critiques.

**Fig 8:**
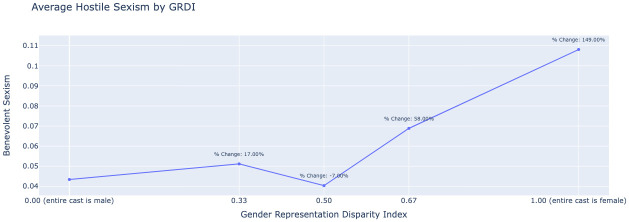
Relationship between the Gender Representation Disparity Index (GRDI) and average hostile sexism in film criticism, illustrating increased sexism as the GRDI moves toward a female-dominated cast.

Notably, the percent change in benevolent sexism from an all-male cast to an all-female cast is 44.00%, a substantial increase. Similarly to benevolent sexism, Fig 8 shows a positive correlation between the GRDI and average hostile sexism. The trend is more pronounced here, with a more evident increase of 149.00% from an all-male to an all-female cast. This steep increase suggests that as the number of women in film roles increases, the film is likely to be subjected to significantly higher levels of hostile sexism. It is noteworthy to highlight that our analysis is based on the magnitude of the model scores and is not capturing the frequency of each bias type.

**Fig 9:**
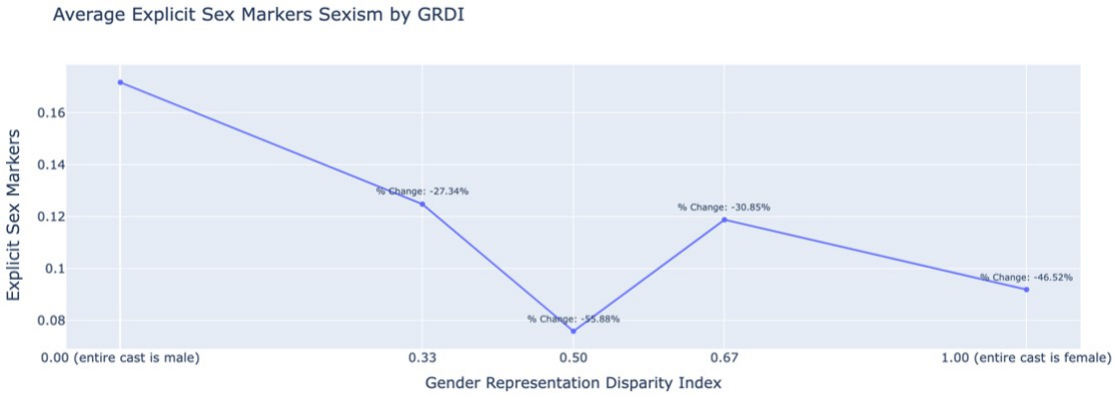
Relationship between the Gender Representation Disparity Index (GRDI) and average explicit sex markers in film criticism, illustrating a decrease in markers as the GRDI moves toward a female-dominated cast.

Fig 9 shows a negative correlation between the Gender Representation Disparity Index (GRDI) and average explicit sex markers in film reviews. As the GRDI moves from an all-male cast (0.00) to an all-female cast (1.00), explicit sex markers decrease by 46.52%. This decrease is likely due to the prevalence of male-specific markers (e.g., ‘businessman’ and ‘cameraman’), which are more applicable in films with predominantly male casts.

#### 5.2.2 Disparity in sexism by movie role and gender

**Fig 10:**
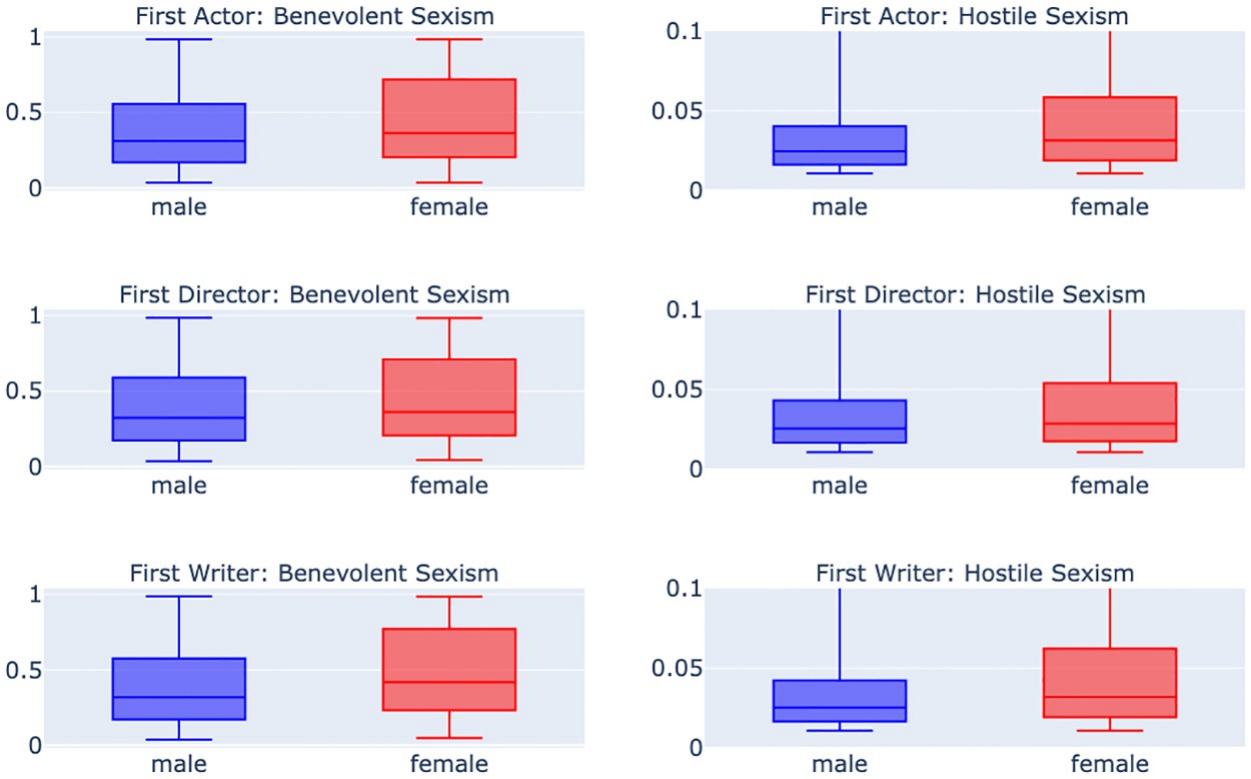
Comparison of boxplots for benevolent and hostile sexism scores in movie review transcripts, segmented by the gender of the first actor, director, and writer, highlighting the differences in antagonism and bias when females lead movie roles.

Fig 10 compares the mean levels of benevolent and hostile sexism based on the gender of the first actor, director, and writer. Both benevolent sexism and hostile sexism are higher when the first actor is female. This pattern also holds true across the director and writer categories, albeit with varying degrees of difference. The difference in hostile sexism between male and female first actors and writers is particularly striking, suggesting that a movie led by a female star actress receives more antagonistic reviews than male-led movies, directly impacting the movie ratings and the actresses’ subsequent earnings.

#### 5.2.3 Disparity in sexism by genre

**Fig 11:**
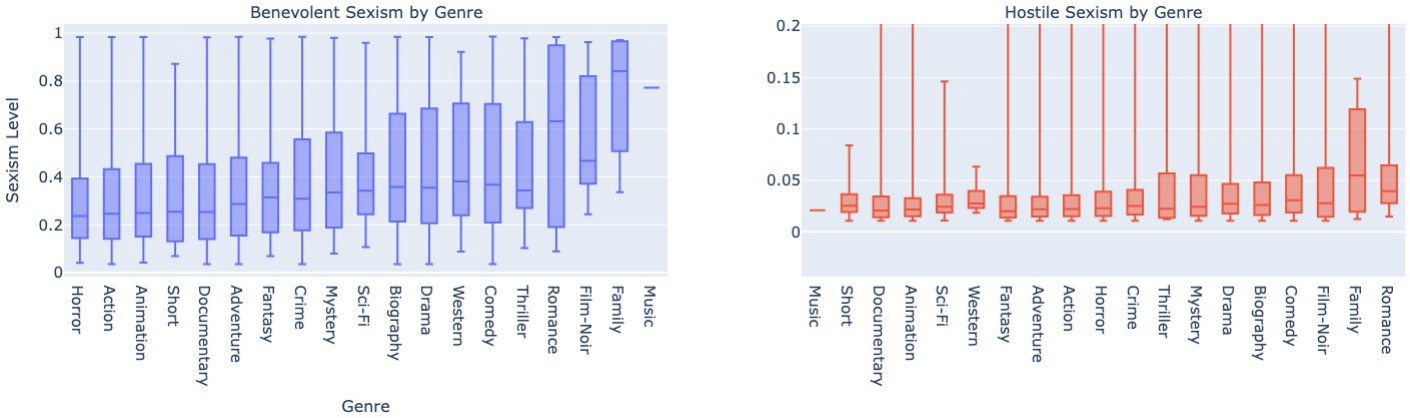
Boxplot comparison of benevolent and hostile sexism scores in movie review transcripts, categorized by movie genres.

Analyzing Fig 11, it is observable in the left plot that benevolent sexism is more prevalent in certain genres. Family and Romance genres show the highest mean levels, which might reflect traditional gender roles being more pronounced in these categories. On the right plot, hostile sexism appears less prevalent overall but shows a notable peak in the Romance genre, suggesting a trend where movies that center on romantic themes might be subject to more critical and antagonistic movie review transcripts. The difference in scale between the two plots is also telling; benevolent sexism levels are significantly higher than those of hostile sexism, indicating that subtle, patronizing attitudes are more common in the film industry than outright hostility. This could be reflective of a broader societal trend where sexism is often masked as protective or complementary, particularly in media representations.

#### 5.2.4 Statistical analysis of gender impact on movie criticism

To investigate the impact of the gender of movie cast members on movie criticism, we analyzed two response variables: *benevolent_ sexism* and *hostile_ sexism*. We considered the gender of the first actor, first director, and first writer for each movie. Given the imbalanced and non-normal distribution of our data, as shown in Fig 11, which may be attributed to clustered scoring patterns and potential outliers, we opted for a non-parametric approach. Specifically, we utilized the Mann-Whitney U test to compare score distributions between male and female categories within each gender-related column.

*First actor.* Significant differences between male and female first actors were observed in the *benevolent_sexism* and *hostile_sexism* variables. The *benevolent_sexism* variable showed a p-value of 6.61 × 10^−42^ and the *hostile_sexism* variable showed a p-value of 1.43 × 10^−67^, indicating a strong bias wherein female first actors tend to receive more benevolent and hostile criticism compared to their male counterparts. The median and mean values for both sexism dimensions were higher for females, emphasizing the existence of gender bias in movie reviews.

*First director.* The analysis for first directors indicated significant gender-based differences in *benevolent_sexism*, with a p-value of 7.15 × 10^−13^. Similarly, a significant difference was found in *hostile_sexism*, with a p-value of 3.98 × 10^−8^, suggesting that female directors are subject to more benevolent and hostile criticism than male directors. The data showed higher median and mean values for female directors in both measures of sexism.

*First writer.* For first writers, there was a notable difference in the *benevolent_sexism* and *hostile_sexism* variables, with p-values of 3.84 × 10^−46^ and 1.46 × 10^−35^, respectively. This implies that female writers are more likely to be the target of both benevolent and hostile sexism. The higher median and mean values for female writers support this indication of gender bias in the criticism of their work.

## 6 Discussion

Our findings reveal significant gender biases in professional movie reviews, with women underrepresented in key roles: only 28% of first-billed actors, 9% of directors, and 14% of writers are female. This disparity limits narrative diversity and reinforces male-centric perspectives within the film industry. This trend aligns with other studies; for instance, in early 2022, male critics accounted for 69% of reviewers, with female critics comprising just 31% and nonbinary individuals a mere 0.3% [38].

Moreover, movies with more women in prominent roles receive higher levels of benevolent sexism, which involve patronizing or stereotypical remarks, as well as hostile sexism marked by overtly negative attitudes in reviews. The magnitude of benevolent sexism scores was 44% higher, while that of hostile sexism was 149% higher in reviews of female-led films compared to male-led films. This suggests that critics may unconsciously judge movies differently based on the gender of those involved, affecting the films’ reception and the careers of female professionals. Our analysis also reveals that as the GRDI shifts from an all-male cast (0.00) to an all-female cast (1.00), the average number of explicit sex markers decreases by 46.52%. This decline is likely due to the prevalence of male-specific markers like ‘businessman’ and ‘cameraman,’ which are more applicable in films with predominantly male casts.

The generic pronouns detected by the model were inapplicable within the context of movie reviews. In movie reviews, when a reviewer uses a gendered pronoun, it typically refers to a specific character whose gender is already established, assuming the reader is familiar with the film’s cast. Hence, this will yield a high false positive rate but would not signify any bias by the reviewer. For example, in the sentence, “He always saves the day in these kinds of movies,” the classifier may interpret ‘He’ as a generic reference to a male action hero. However, within the context of a specific movie review, ‘He’ likely refers to the main character, whose gender is established within the narrative.

Additionally, we found that instances of predictions of dehumanization in the movie reviews were not significant enough to include in the results. Such language is often reflected in film narratives, where female characters are portrayed in traditional or dependent roles. Reviewers discussing these portrayals will use dehumanizing language, but this stems from the content of the films rather than the critics’ own biases. Therefore, most positive detections were due to narrative bias, not review bias. In future work, we aim to differentiate between dehumanization originating from the film’s storyline and that from the reviewers themselves to better understand this dynamic.

### 6.1 Impact of sexist reviews on women in the film industry

Gender biases can deter women from pursuing careers in the film industry and shape audience perceptions. The harmful impact of sexist reviews on women in film is far-reaching, influencing career paths, financial security, mental well-being, and the broader landscape of media representation.

#### Implications of high GRDI

Films with a high GRDI indicates strong female representation and often feature diverse female roles that break away from stereotypes, challenging ingrained gender norms. Films featuring complex female characters can trigger discomfort among audiences and critics accustomed to traditional gender roles, leading to more critical reviews. This backlash is particularly noticeable in Hollywood, where traditional expectations around female roles are deeply ingrained [39, 40]. For instance, a film with an almost all-female cast may inherently subvert conventional depictions, presenting female characters with depth and agency. However, these portrayals may provoke resistance from critics whose biases favor conventional portrayals of women. This could explain why films with a high GRDI might attract more negative sentiment, as they disrupt longstanding norms and challenge conventional expectations of gender roles [39, 40].

#### Impact on career opportunities

Negative reviews often overshadow women’s performances, leading to diminished visibility and fewer opportunities for future roles. This lack of recognition can hinder career advancement and perpetuate gender disparities in the industry. Research indicates that women and people of color are disproportionately chosen to work for movies in lower-grossing categories, potentially affecting their visibility and recognition [41]. Sexist critiques can reinforce narrow perceptions of female talent, leading to stereotyping. Women may be pigeonholed into specific roles that conform to traditional gender norms, limiting their ability to explore diverse characters and narratives. Studies have shown that male characters are given more agency than female characters, which can influence the types of roles available to women [42]. Female writers and directors create films with more balanced gender ratios in characters than their male counterparts [43]. Additionally, female characters tend to have more positive utterances, while male characters use more achievement-related language. Male characters are also more likely to use words associated with death and swear words compared to female characters [43].

#### Financial implications

Negative reviews can significantly influence a film’s box office performance, affecting the financial prospects of all involved, including actors, directors, and writers. For women already underrepresented in key creative roles, the financial repercussions of poor reviews can exacerbate existing income disparities in the industry. The average domestic box office revenue of a film by a female director was 37% less than that of a male-directed film, suggesting financial implications for female filmmakers [41]. Furthermore, actors and filmmakers often rely on positive critical reception to secure back-end deals or participate in profitable projects. Sexist reviews can diminish a film’s commercial viability, reducing opportunities for financial gain in subsequent roles. The underwhelming performance of films directed by women may stem from selection bias by Hollywood studios, which could impact future opportunities [41].

#### Psychological and emotional consequences

The pressure from negative critiques, particularly those laden with sexism, can lead to heightened stress and anxiety levels among female industry professionals. Public scrutiny and social media commentary can exacerbate this mental toll, creating a hostile work environment that affects overall job satisfaction and performance. The prevalence of harassment and discrimination in the film industry contributes to a toxic work environment, impacting mental health. This is exacerbated when films contain sexist elements, as these critiques can be more personal and targeted. A study indicated that 87% of respondents in the UK film and TV industry reported experiencing mental health issues, with 64% experiencing depression, which is notably higher than national averages [44]. Furthermore, continuous exposure to sexist commentary can lead to self-doubt among women, where they may internalize the negative perceptions propagated by critics. This can hinder their confidence in their abilities and diminish their willingness to take creative risks or pursue leadership roles. The underrepresentation of women in key creative roles can lead to internalized biases, affecting their confidence and opportunities [45].

#### Perpetuation of gender inequality

Sexist reviews not only affect individual women but also perpetuate broader societal biases against female representation in media. When critical language marginalizes women, it reinforces stereotypes that undermine their contributions, thus perpetuating a cycle of gender inequality within the industry. Nevertheless, the negative framing of female-led films can influence audience perceptions, leading to a cultural narrative that devalues stories featuring women. This can result in reduced interest in female-centric narratives, further limiting opportunities for women in filmmaking and storytelling. The portrayal of women in films often reflects and reinforces societal gender biases, affecting audience perceptions [42].

#### Long-term effects on industry culture

Sexist critiques contribute to an industry culture that may discourage aspiring female filmmakers, writers, and actors from pursuing careers in film. When the environment is perceived as hostile or dismissive, potential talent may opt for other industries, leading to a lack of diversity and innovation in storytelling. The underrepresentation of women in the film industry can create barriers to entry for aspiring female professionals [45]. As long as sexist reviews continue to influence industry standards, progress toward gender equity in the film industry will be hindered. A culture that tolerates or ignores sexism fails to acknowledge the importance of diverse voices and perspectives, hindering the evolution of storytelling. The persistence of gender inequality in the film industry can hinder progress and innovation [45].

#### Dual impact of gender representation and reviewer bias

The results from our study highlight two concurrent issues within the movie review system. On one hand, men and women are often cast in roles that align with stereotypical gender expectations, which reinforces these norms. Audiences and critics frequently show a preference for traditional gender roles, reinforcing gendered expectations through both praise and critique [39]. On the other hand, critics bring their own gender biases to their assessments, carrying implicit assumptions about gender that shape their language and evaluations. These biases often serve to reinforce conventional portrayals and critique films that challenge established norms [40]. This duality creates an environment where films may face biased reviews whether they adhere to or challenge gender norms.

### 6.2 Improving gender balance in the film industry

Our findings underscore the substantial impact of gender bias in movie reviews, revealing how it influences both the perception of female-led films and the representation of women in the industry. The film industry can take actionable steps to improve gender balance and create more inclusive, equitable storytelling. Our analysis demonstrates that female-dominated casts receive higher levels of both benevolent and hostile sexism in reviews, with female characters more often framed in traditional or dependent roles. Recognizing these biases can inform industry professionals, encouraging more conscious storytelling decisions that avoid harmful stereotypes and promote gender-equitable narratives.

Professional critics significantly influence public perceptions of films and can either reinforce or challenge gender biases. Our study reveals that reviews of female-led films often contain both benevolent and hostile sexism, impacting audience attitudes and contributing to the marginalization of women in the industry. To promote more balanced and unbiased criticism, critics should recognize inherent biases in their critiques to avoid language that reinforces gender stereotypes or diminishes female-led projects. Awareness can lead to more deliberate language choices that evaluate films on merit without unconsciously reinforcing gendered expectations. Developing training sessions and workshops can help critics identify and mitigate gender biases in their writing. Such initiatives, organized by journalism schools or film festivals, for instance, can assist critics in recognizing how certain language patterns contribute to a skewed view of female characters and creators.

Providing fair and unbiased evaluations supports a more diverse and inclusive industry, encouraging studios and audiences to appreciate a wider variety of narratives. The underrepresentation of female critics can lead to a lack of diverse perspectives, affecting industry standards. Increasing the diversity of professional critics, including more women and underrepresented voices, can help reduce the prevalence of gender bias in reviews. Diverse perspectives lead to more nuanced and inclusive critiques, challenging the industry’s traditional standards. Studies have found that a more diverse group of critics can provide a broader range of perspectives, leading to more balanced reviews [22].

Awareness of gender biases in reviews can prompt filmmakers to rethink portrayals of women, leading to richer, multi-dimensional female characters that break away from stereotypical roles. The film industry is beginning to address gender imbalance with initiatives like the 50/50 by 2020 campaign [46] and gender-focused production companies. Some studios have started hiring consultants to assess representation in scripts, helping to build more balanced characters. Additionally, film festivals and award committees increasingly recognize and promote films with diverse casts and crews.

To build on these efforts, studios can adopt guidelines and benchmarks for gender representation in key production roles, including directors, writers, and lead actors. Establishing mentorship and funding programs specifically for women and underrepresented groups can help build an inclusive industry. Moreover, adopting gender parity requirements for film festivals and major awards could further motivate studios to prioritize diverse hiring practices.

The film industry has long relied on traditional narratives, and breaking away from them poses cultural and financial challenges. There can be hesitation to shift away from familiar tropes, particularly in genres like romantic comedies and family films, which often perpetuate gender stereotypes that audiences have grown accustomed to. Financial incentives often drive studios to follow familiar, established narratives that are perceived as “safe.” This pressure to generate profit can reinforce the use of formulaic plots that perpetuate harmful gender biases. Financing remains a barrier for stories that deviate from traditional plots. Films led by women, or those that explore complex female characters, often receive lower budgets or less marketing support than their male-dominated counterparts, limiting their visibility and reach.

Consumers play a crucial role in shaping industry trends. By choosing to support films that promote gender balance and challenge stereotypical narratives, audiences signal to studios that there is a demand for more inclusive content. While there is an undeniable demand for nostalgic narratives, including those prevalent in classic Disney movies, it is essential to challenge the notion that audiences won’t accept more progressive storytelling. Many viewers appreciate updated takes on traditional stories that reflect contemporary values, offering familiar elements without perpetuating outdated stereotypes.

### 6.3 Limitations

Our study presents several limitations that should be considered when interpreting the findings. First, because our dataset primarily consists of Western films and reviews by Western critics, the biases observed may be specific to Western cinema. This focus may limit the generalization of our results to a global context, as cultural variations in gender representation and criticism could yield different outcomes. Future research would benefit from a more diverse sample, incorporating films and reviews from multiple cultural backgrounds to capture a broader spectrum of industry practices.

Second, our study could not account for the demographics of the reviewers, such as gender and age, due to a lack of available metadata. Reviewer demographics are significant, as characteristics like gender or generational background may influence perceptions of gender in film and the type of language used in critiques. Including this metadata in future studies would allow for a more nuanced analysis of how personal biases intersect with professional critique.

Additionally, we limited our analysis to the first-billed actor, director, and writer, which may not fully represent gender diversity in the entire cast and crew. While this approach preserves the GRDI by focusing on lead roles, it may overlook biases affecting the broader cast. Expanding the analysis to encompass the entire cast and crew could provide a more holistic understanding of gender representation. Future research should explore the effects of different combinations of female roles, assess the impact of role visibility on gender bias through mention frequency, and examine how the severity of sexist backlash varies across professions, especially for high-visibility roles like directors.

Our gender identification method also relied on name-based predictions, which do not recognize non-binary or gender-diverse individuals. Future studies should incorporate more inclusive gender identification practices, enabling a broader analysis of gender representation.

Finally, our analysis may conflate biases originating from the film’s narrative with those introduced by the reviewers. Critics often comment on or critique portrayals within the film, which can introduce biased language that reflects the film’s content rather than the reviewer’s personal biases. Future work should seek to differentiate narrative-driven biases from those inherent to the reviewers’ perspectives, allowing for a more precise assessment of the reviewers’ biases. We also aim to explore role-specific and entity-based gender bias classification, which could enable us to analyze bias at the level of individual characters and roles.

## 7 Conclusion

This work explored the prevalence of biases within the film industry, particularly focusing on linguistic biases in professionally written reviews. To do so, we created a metadata-enriched movie review dataset that includes 17,165 review transcripts and metadata on the gender of each reviewed movie’s first actor, director, and writer. We then employed our gender bias detection system to measure various biases within review transcripts. Our findings revealed severe under-representation of women in key film roles: 72% of first actors, 91% of first directors, and 86% of first writers being male. Moreover, films with predominantly female casts faced 44% more benevolent sexism and a 149% increase in hostile sexism in reviews compared to male-dominated casts, a trend consistent across genres. Our analysis highlights the pervasive biases in the film industry, underscoring the need for more equitable and unbiased representation.

In conclusion, professional critics have the opportunity to drive positive change in the film industry by embracing more balanced language and supporting diverse storytelling. These actions can help dismantle harmful narratives that reinforce gender biases, leading to a richer, more inclusive cinematic landscape. Encouraging critics to challenge traditional narratives and advocate for more nuanced portrayals of female characters can lead to a richer, more inclusive film landscape that genuinely reflects diverse experiences and perspectives. By respecting and valuing feminist film criticism, the industry can move toward a more equitable and inclusive environment.

## 8 Appendix

### A review example

Rob Roy (1991)

by Pedro Sena

This is a much better film than most people expect. And unlike many films that show a lot of class, this film also adds a tremendous showcase of acting, that is exceptional. One need only watch Tim Roth, in an exceptional performance as the bad **guy** in this film, to recognize that indeed this is an Oscar level performance, and extremely well deserving of its nomination. Winning it will depend on the amount of favoritism for home town *actors*. ROB ROY is the story of an Irish man, who became a folk hero and leader of a large group of Irish people, with whom various kings had some problems. And as is usually the case, there were many instances where there are political interests that interfere with the people’s plans, if not the king’s. In this case, one of the king’s best **men**, has become a bit of an enemy, and spends the whole film chasing, and finally getting a chance to meet his nemesis. In between, we get all the intrigues that make this story worth while, and special. **Rob Roy’s wife gets raped**, and most of his village gets destroyed. But in the long run the honest prevail in this tale between good ad evil, and whose side is the right one, the law, or the people. This is a very well directed film, at least it is done with much care and attention to detail, and while the main character, played by Liam Neeson, does not display much of the definition and attention to detail that all others do, he at least tries to hold his own. A lot of it may just be a struggle with the dialect, but all in all he does alright…. it might have been easier to have cast someone local, with a better handle on the language to get a stronger film. But Liam is not bad, although one gets the feeling that he is struggling with his lines at times. By comparison, the work of Tim Roth as the villain, and John Hurt as the king, are much smoother and well done. In many ways this is the way Hollywood likes it. **A good looking man to attract the ladies, and the rest a cast of magnificent loons that can hold just about any script they are given.** Tim Roth’s work, is just way above and beyond all others, and stands out as the single element that moves this film as far as it does, and creates a more powerful conclusion to the epic. With some magnificent cinematography, displaying the virgin country sides, this is a very pretty film to see, although it has its moments where the violence is a bit too strong. But, the director keeps these moments to the minimum. Excellent film all around. Worth seeing. Well directed. One word…. Tim Roth.

### B examples with high gender bias score

#### Example with high score for benevolent sexism

Women exist here to look good, comfort the man, and get argued over.

She may be brilliant for a woman, but she still doesn’t hold a candle to her male counterparts.

Because he’s the type of guy that men want to be, and she is the girl that most women secretly wish they were (but will never ever admit ever).

#### Examples with high score for hostile sexism

The worst kind of woman in my book, the type that knows they’re gorgeous, uses it, and then dumps all over you when you show the least bit of interest.”

Maybe we’ll put a gender spin on it, see if we can get some women out to see it.

He tells her that even though women want to be respected, they want to be taken by a man who desires them, and a stranger usually gets to her first, as a friend is too polite to act on his impulses.
